# Meeting of the Northeastern Indiana Medical Society

**Published:** 1873-01-01

**Authors:** John Dancer, J. L. Gilbert

**Affiliations:** Pres.; Sec’y.


### Society Reports.

### MEETING OF THE NORTHEASTERN INDIANA MEDICAL SOCIETY.

Tuesday, December 3d, 10 o'clock A. M.—The meeting was held at Ligonier, in the office of Dr. Knepper, Dr. Dancer presiding.

Members present—Drs. Cowan, Spooner, Casebeer, Chamberlain, Wood, Dancer, Denny, Palmiter, Knepper, Crum, Landon, Gants, Roehdebaugh, Franks, Carr, Latta, Erickson and Gilbert.

After some preliminary business, Dr. Franks reported the following case: Patient aged 20; male; in good health, with the exception of an attack of epilepsy, which recurs about once a year. As yet it has resulted in no visible constitutional disturbance. During a violent attack on the 29th day of last August, he fell from a chair. After the attack he complained of considerable pain in the left shoulder. A physician was called, who examined the shoulder, but found no displacement. He prescribed liniment, and gave other directions. About four weeks afterward, the patient applied to Dr. Franks, who found a downward dislocation of the humerus. The patient was brought under the influence of chloroform, and the luxation replaced, with the aid of Dr. Gard. It was the opinion of Dr. Gard (who was called immediately after the attack) that the luxation was the result of continuous muscular action, and not the result of violent muscular action during the attack, or from the fall. This opinion was not fully concurred in by the Society. Dr. Denny, however, believed a luxation might be possible from continuous muscular action, although in this case it probably occurred during the attack.

Dr. Cowan reported a case of periodical epistaxis in a child aged eight years—healthy parentage. The patient was put on iron and the mineral acids, besides periodic doses of quinea, nutritious diet, bathing, etc. This treatment was continued for some time, with no improvement. At length an eruption on the skin occurred, with coppery discoloration. The doctor believing it to be of a syphilitic character, prescribed "Donovan's Solution," which resulted in a speedy recovery.

Dr. Landon presented a case—female, aged fifteen—of cancerous cachexy. The patient has a vascular tumor under the tongue, near the orifice of Wharton's duct, and one on the cheek, near the opening of Steno's duct, which has a hard consistency, but not of the nature of concretions from the saliva. The neck, externally, presents a few varicose veins.

Dr. Latta—The tumor under the tongue is varicose, of course; but owing to its location I would consider its removal very dangerous, on account of the hæmorrhage which might prove uncontrollable. If I were to operate at all, I should destroy the vessel by injecting it with sol. ferri persulphas. I would use as large a quantity of the solution as possible, as I believe there is less danger from clot when the inflammation produced is considerable, than when it is but slight, for obvious reasons.

Dr. Erickson—I should use the "Vienna paste" in destroying vascular tumors. The danger from clot is very slight indeed with the paste. I have frequently used it in destroying tumors of this kind, and have never experienced bad results. I should consider an operation extremely hazardous in this case, however, on account of its location.

Dr. Denny—I believe it to be cancerous, and would advise no interference.

Dr. Erickson presented a calculus, measuring two inches in circumference, which he removed from the urethra of a gentleman three days before. It had passed from the bladder and lodged in the urethra about an inch below the prostate gland. It was impossible to remove it except by the knife. The patient bids fair to make a good recovery.

A chemical analysis shows the calculus to consist of oxalate of lime.

Dr. Crum reported a case: Girl aged two years. In falling upon the sharp corner of an open cellar door, the walls of the abdomen were broken through and the colon was ruptured, the bowels protruding through the aperture. The wound was dressed with the assistance of Dr. Knepper. The patient was put on opium, and complete quiet was observed. A good recovery was the result.

Dr. Latta spoke of the importance of full doses of opium in such cases, and of the necessity of avoiding cathartics. He would rather allow the bowels to remain constipated for a month than give a cathartic in such a case.

Dr. Chamberlain reported a similar case, which also resulted in recovery. A woman, in attempting to commit suicide, had opened the abdomen with a knife, near the umbilicus, and withdrawing the colon, had cut away about four inches of it. The ends were returned and the wound dressed. The doctor gave a full dose of opium, and left the patient to die, as he supposed; but to his astonishment she recovered.

Dr., H. D. Wood reported the following case: Male; aged 35; parentage good; entered the army in good health in 1861, and served until 1865. During the summer of 1864 he suffered much from diarrhœa. In the winter of 1864-5 he began to complain of pain in the ball of the left foot when walking. The pain extended to the heel, and thence all over the foot. In 1866 the other foot was involved in the same way. On rising in the morning he suffers no pain, but walking produces pain and a sense of tension. When the feet are elevated there is no pain. At first there was but little swelling, but it gradually increased a little. There is some redness now, and slight pitting on pressure. The affection has been growing more painful from year to year until the present time. He first came under the doctor's treatment a year ago. He has been unable to afford any relief whatever. He now submitted the case to the Society, and asked for an expression of opinion in regard to its pathology. There was an extended discussion in this case, but nothing satisfactory was elicited.

A number of other cases of much interest were reported, and an animated discussion was had on uterine supporters.

T. F. Wood, G. Erickson and P. W. Crum were appointed essayists for the next meeting.

The subject of discussion at the next meeting will be: "Can legislation be Protective to the Medical Profession?"

The Society adjourned to meet at Angola, on the first Tuesday in March, 1873.

John Dancer, *Pres.*

J. L. Gilbert, *Sec'y.*

